# A multidisciplinary effort to increase COVID-19 vaccination among the older adults

**DOI:** 10.3389/fpubh.2022.904161

**Published:** 2022-08-01

**Authors:** Aminath S. Moosa, Yi M. S. Wee, Meng H. Jaw, Qifan F. Tan, Wan L. D. Tse, Chui Y. Loke, Guan L. A. Ee, Chee C. D. Ng, Wai K. Aau, Yi L. E. Koh, Ngiap C. Tan

**Affiliations:** ^1^SingHealth Polyclinics, Singapore, Singapore; ^2^SingHealth-Duke NUS Family Medicine Academic Clinical Programme, Singapore, Singapore

**Keywords:** COVID-19, older adult, COVID-19 vaccination, vaccine promotion, vaccination program, multidisciplinary team

## Abstract

**Background:**

COVID-19 vaccination significantly reduces the risk of infection and its associated morbidity and mortality. However, poor uptake of the COVID-19 vaccination was reported among the high-risk group of older people amidst emerging variants of concern. This community case study reports an outreach program in Singapore, COVE (COVID-19 Vaccination for the Elderly) initiated by healthcare workers in a cluster of primary care clinics. They assessed the vaccine hesitancy among these older persons, addressed their concerns and facilitated their vaccination appointment during a brief phone conversation.

**Method:**

Twenty one thousand six hundred and sixty three unvaccinated adults aged ≥60 years were contacted by healthcare worker volunteers over two phases from June to October 2021. In phase I, they contacted adults aged above 70 years over 2 weeks. Adults who were uncontactable in phase I and those aged 60–69 years were sent SMS in phase II. Data were analyzed *via* descriptive data analysis.

**Results:**

After phase 1, 65.5% (*n* = 5,646/8,617) of older adults had received at least one dose of the COVID-19 vaccine. The respondents expressed intention to vaccinate (39%, *n* = 3,390), requested to seek further information (25%, *n* = 2,138), reported access barrier (8%, *n* = 715), or were concerned of the vaccine adverse effects (3%, *n* = 288). Vaccination was refused by 24% (*n* = 2,086) of the respondents. Eventually 60.4% (*n* = 13,082/21,663) of them were vaccinated 3 months after COVE implementation.

**Conclusion:**

The COVE program increased the COVID-19 vaccination uptake of older adults from 84.6 to 96.3%. A person-centric proactive approach by healthcare workers addressed vaccine hesitancy and optimized vaccination. The outreach scheduling of vaccination appointments is key in promoting vaccination uptake among older adults.

## Introduction

A novel coronavirus now designated SARS-CoV-2, has resulted in a pandemic caused by the Coronavirus Disease 2019 (COVID-19) (1). Approximately 265 million people were infected, and more than 5.2 million deaths were reported globally as of 5 December 2021 (2). In a survey conducted by Nature in January 2021, almost 90% of the immunologists, infectious disease researchers and virologists involved in the research of COVID-19 think the COVID-19 virus will become endemic (3). Since then, many countries, including Singapore, have shifted from their healthcare policies from an elimination strategy to endemicity (4). The disease burden of the COVID-19 virus has been immense and will persist or even escalate if it becomes endemic (5, 6). Vaccination to prevent individuals from getting seriously ill or dying is a key primary prevention strategy against the coronavirus, including the emerging variants of concern (VOC) (7). At the time of writing, 10 COVID-19 vaccines have been approved to be used by the World Health Organization (8). All COVID-19 vaccines are effective and safe against the COVID-19 original strain and the variants of concern, with the mRNA vaccine having the highest efficacy in preventing symptomatic cases of COVID-19 (8). In older adults, vaccination against COVID-19 reduces symptomatic COVID-19 and offers protection against severe disease (9).

Vulnerable groups such as older adults are at higher risk of severe COVID-19 morbidity and mortality (10). The case fatality rate and susceptibility to symptomatic COVID-19 are higher in the older adult (11). Based on the Korea Center for Disease Control and Prevention report in January 2022, the overall case fatality rate (CFR) was 0.91% among 705,905 confirmed cases. However, the CFRs were much higher in the older adult at 4.38 and 14.45% in the 70–79 and 80 years age groups, respectively (12). The CFR were 8.0 and 14.8% in similar age groups in infected older adults in China when the overall CFR was 2.3% in February 2020 (13). Locally, among cases aged 60–69 years and 70–79 years, the age-specific CFRs in April 2020 were estimated as 1.84% (95% confidence interval: 0.46–4.72%) and 5.57% (1.41–13.97%), respectively (14), when the overall CFR was 0.2% for the same period (15). As the older adults have significantly higher mortality rates after COVID-19 infection, they have been the prime target recipients of the vaccines (16, 17).

Nation-wide campaigns were rolled-out to encourage older adults to get vaccinated against COVID-19. In Singapore, COVID-19 vaccination for older adults aged 70 years and above started in late January 2021, and in mid-March 2021 for adults more than 60 years old (15). Media reports in the country's four official languages and local dialects and COVID-19 vaccine promotional messages were regularly delivered by public figures and politicians to the public. The Singapore government sent short message service (SMS) texts to older adults to promote vaccination and facilitate vaccination booking *via* online links. Incentives in the form of vouchers were provided to families to encourage their older family members to get vaccinated (18, 19). With these interventions, there was a rapid increase in vaccine uptake from February to March 2021. However, a local study noted a decline in vaccination uptake from May to June 2021 (20). By 28 July 2021, about 28 and 18% of adults aged 70 years above and 60–69 years, respectively, had yet to be fully vaccinated against COVID-19 (21).

A recent systematic review and meta-analysis, which included 15 (22) studies from Asia, Europe, and the USA, reported that 19% of older adults were hesitant to take COVID-19 vaccine (23). Vaccine hesitancy is influenced by multiple factors such as confidence in the development, safety and efficacy of the vaccine; confidence in the vaccination center or provider; complacency in acknowledging the severity of the disease and convenience (24). Tan et al. reported that perception of side effects of the available vaccines was a major concern that deterred the unvaccinated older adults (20). The provision of information on the safety and effectiveness of vaccines by healthcare providers can potentially boost their confidence in the vaccine uptake (25). A cross-sectional study in China reported that addressing perceived barriers and strong recommendations from authorities, including health care providers, would promote COVID-19 vaccination among local Chinese (26).

To accelerate the uptake of the COVID-19 vaccination program in Singapore, a potential approach was to leverage on the primary care provider's long-standing relationship with older adult patients to nudge them to be vaccinated. The success of such an initiative hinge on the clarity and ability of healthcare workers to deliver the information to these older adults in languages or dialects understood by them, especially in the multi-lingual, multi-ethnic Asian population in Singapore. Evidence has shown that contextualized and culturally adapted interventions are effective to build trust, enhance confidence and mitigate vaccine hesitancy (22).

The COVE (**C**OVID-19 **V**accination for the **E**lderly) is designed as an outreach program in one of the three clusters of public primary care clinics (polyclinics) in Singapore, SingHealth Polyclinics (SHP). Its healthcare support staff were trained to contact the older adults and provide them with information relating to the COVID-19 vaccination. Ultimately, the objective of the COVE program was to increase the vaccine uptake among these older adults against the coronavirus.

Hence, this community study aimed to determine the reasons for the delay in COVID-19 vaccination among older persons managed in polyclinics and their subsequent uptake of the vaccine after a brief phone conversation.

## Methods

### Setting and study population

SingHealth Polyclinics (SHP) is a network of eight subsidized public primary care facilities located in the eastern region of the island state (27). It is an accredited Family Medicine training center in Singapore. SHP managed 4.2 million patient attendances in 2020 based on the institution's electronic medical record (EMR) system and business database. Over 199,000 adults aged 60 years and older contributed to 30–42% of the patient attendances in each of its polyclinics.

### Data collection and intervention

SHP's Healthcare Information Unit extracted baseline data from SingHealth-Integrated Health Information Systems Electronic Health Intelligence System (eHINTs) on 29 July 2021. COVID-19 vaccination data is collated *via* a national health portal and automatically flows to the eHINTS system. A list of unvaccinated adults aged 60 years and older who consulted any of the eight polyclinics during 2020 and 2021 on 27 July 2021 was generated. Additional data on patient demographic characteristics and contact numbers were derived from Outpatient Administrative Systems (OAS).

### Preparation for the launch of the COVE program

The COVE program was proposed by SHP's Chief Executive Officer, developed by the leaders from the Clinical Services and Operations departments and executed by a steering team in collaboration with volunteers among the multidisciplinary staff at its headquarters (Clinical Services, Operations, Finance, Human Resource, Nursing, Quality Management, Polyclinic Development, Allied Health, Medical Informatics, Education and Research).

On 3 August 2021, the steering team organized and briefed volunteer representatives from all departments in SHP headquarters. The team provided the COVE program details and the tools required for the intervention. The tools included a call list, excel tracking sheets, call scripts and information on vaccination. Queries and doubts were clarified during this briefing session. In turn, the representatives shared the intervention details and tools with colleagues from their respective departments. A total of 178 staff from the 19 departments participated in the COVE program over two phases.

### COVE program

The program was designed to be implemented in two phases to space out the COVID-19 vaccination of these older adults should they decide to proceed with it. Such deliberate measures would avoid crowding and minimize cross-infection at the vaccination centers, including those in the polyclinics.

### Phase I

In the first phase, the program targeted unvaccinated older adults aged 70 years and above. The rostered staff called them personally *via* phone for 2 weeks, from 4 August 2021 to 20 August 2021. The staff assessed the older adults' intent to vaccinate, their concerns and the need for further information. They advised on vaccination benefits, venues available for vaccination and arranged appointments for older adults who were keen to vaccinate at the polyclinics. The older adults' responses were documented by the staff who updated the number of respondents daily and reported the information to the steering team every week during the intervention period. Respondents with clinical concerns or mobility issues were directed to the clinicians to address their concerns or provide information on alternative venues for vaccination.

A tailored reminder message was developed by the institution and was embedded in the EMR system from 6 Aug 2021 to alert the healthcare workers to unvaccinated patients during their consultation. The alert consisted of a prompt stating “Patient has no record of COVID-19 vaccination. Offer vaccination if eligible.” The alert was triggered when the clinicians (including polyclinic physicians, nurse clinicians, pharmacists) accessed the clinical document of unvaccinated adults. The message reminded the clinicians to review their patients COVID-19 vaccination status and to offer the free vaccine.

### Phase II

Phase II targeted adults aged 60–69 years (*n* = 8,145) and those who were uncontactable after three attempts in phase I. SMS messages were sent out on 18 August 2021 to adults in this phase. Letters were progressively mailed to those aged 70 years and above after 24 August 2021.

### Post-intervention data collection

The vaccination status of targeted adults was collated weekly by the steering team from the EMR system to monitor the program progress. The data also included their response to the phone calls and change in vaccination status post-intervention. The vaccination status was reviewed after 3 months of initiation of the COVE program on 27 October 2021. Additional data such as nationality, marital status, local social and healthcare financial assistance status, such as the Community Health Assist Scheme (CHAS) and Medifund, were traced (28). Medifund is an endowment fund set up by the Government of Singapore. Community Health Assist Scheme is a tiered health financial scheme with different levels of subsidy based on the household incomes; CHAS green, CHAS blue and CHAS orange with monthly household income per person above SGD 2,000, SGD 1,201 to 2,000, and SGD 1,200 and below, respectively.

### Data analysis

An SHP Health Information Unit staff deidentified the collated data, which was subsequently analyzed by a data analyst from the Research department. Descriptive statistics for the patient demographics, response to the audio phone calls and total population vaccinated over time were computed.

### Ethics consideration

An ethical review by Institutional Review Board is not required for this reporting as all data are de-identified and anonymous. Administrative approval from SHP has been obtained to publish the data from the COVE program.

## Results

The SHP registry had noted 21,663 (11%) of 199,218 older adults were unvaccinated. Table 1 shows the sociodemographic characteristics of the unvaccinated adults by age. About two-thirds (62.3%) of them were older at 70 years of age and beyond. More older adults aged ≥70 years (92%) had non-communicable diseases such as hypertension, dyslipidemia and type-2 diabetes mellitus as compared to those aged 60–69 years (78.9%).

**Table 1:** Sociodemographic characteristics of the unvaccinated adults by age groups.

**Demographic characteristics**	**Total unvaccinated patients**	**60–69 years old**	**≥70 years old**
*N* (%)	21,663 (100%)	8,145 (37.6%)	13,518 (62.3%)
Female (%)	42.6%	47.3%	39.8%
**Race (%)**		
Chinese	80.0%	75.6%	82.0%
Malay	9.4%	11.2%	8.4%
Indian	7.0%	8.5%	6.1%
Others	4.0%	4.6%	3.6%
**CHAS**^a^ **(%)**		
No CHAS	32.0%	35.9%	30.0%
CHAS Green^b^	6.9%	8.5%	5.9%
CHAS Orange^c^	14.2%	16.3%	13.0%
CHAS Blue^d^	46.9%	39.3%	51.5%
Presence of Medifund^e^	13.1%	15.6%	11.6%
Presence of Non-communicable Disease (%)	87.0%	78.9%	92.0%
Influenza vaccinated	5.6%	3.7%	6.7%

a *Community Health Assist Scheme = Health financial scheme with different levels of subsidy;*

b *Household monthly income per person above SGD 2,000;*

c *Household monthly income per person SGD 1,201–2,000;*

d *Household monthly income per person SGD 1,200 and below;*

e *An endowment fund set up by the Government of Singapore*.

**Figure 1:**
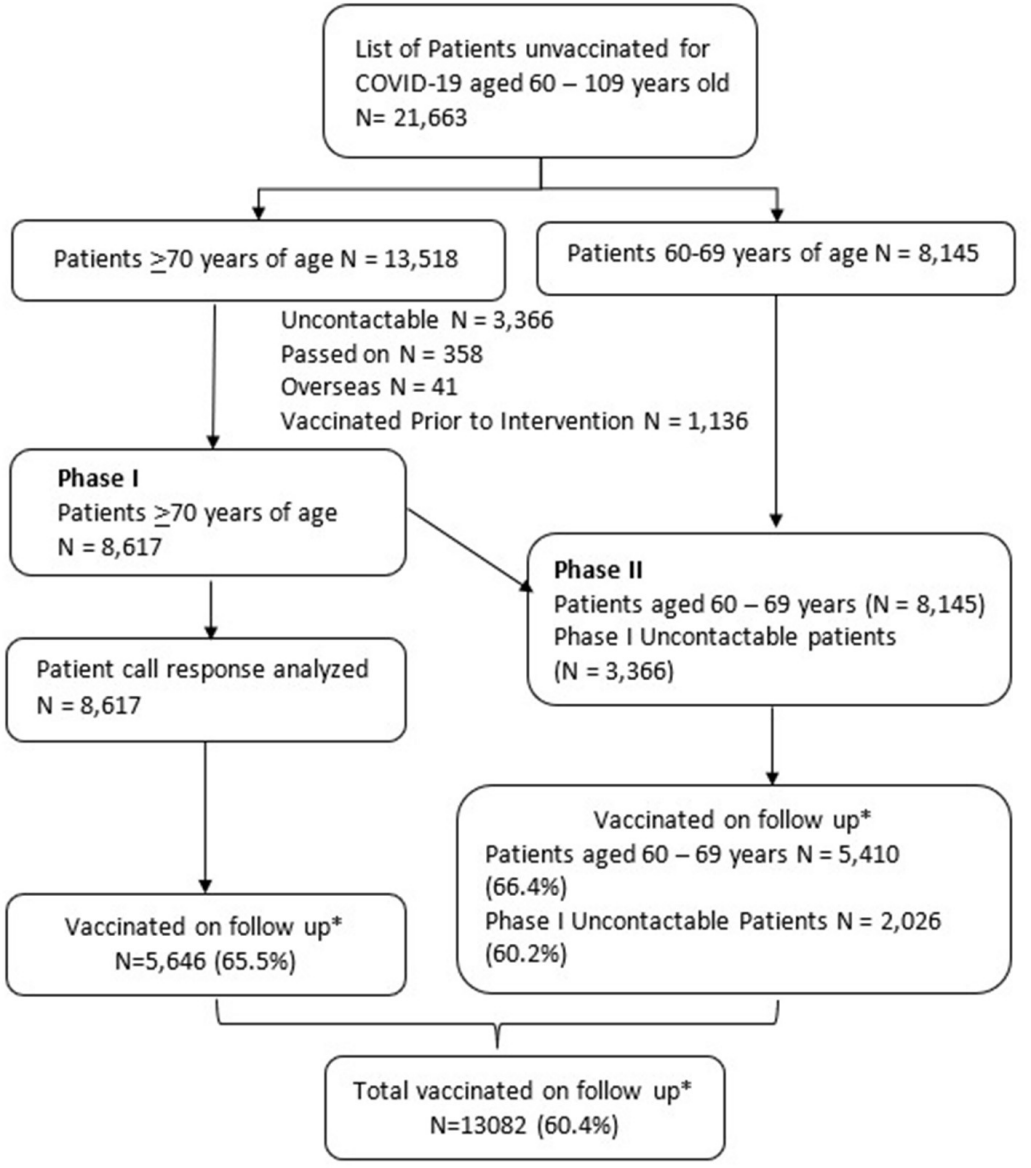
Flow chart for COVE (COVID-19 Vaccination in Elderly) program phases I and II. *Follow up done on 27 October 2021 (3 months after COVE^1^ initiation). ^1^COVE, COVID-19 Vaccination in Elderly.

At commencement, 1,136 adults in the generated list had been vaccinated and hence were excluded from the data analysis. At the end of phase I, staff failed to contact 3,366 of the 13,518 adults; 357 had passed on, and 41 were overseas (Figure 1).

Among the 8,617 older adults contacted *via* phone, their responses were categorized into five main responses. Their subsequent vaccination uptake was reviewed 3 months after phase I, on 27 October 2021 (Table 2).

**Table 2:** Patient call response and vaccination uptake on follow up for phase I patient population.

**Patient call response**	**Total call population *n =* 8,617 *n* (%)**	Total vaccination on follow up^*^n = 5,646 (65.5%) *n* (% vaccination per response)
Self-reported intend to vaccinate	3,390 (39%)	2,760 (81%)
Express desire to seek vaccine information	2,138 (25%)	1,437 (67%)
Outright reject vaccination	2,086 (24%)	851 (41%)
Perceived barrier to access	715 (8%)	455 (64%)
Perceived risk of adverse effect of vaccination	288 (3%)	144 (50%)

* *Follow up done 3 months after phase I on 27 October 2021*.

The majority of the older adults (39%, *n* = 3,390) self-reported they had intended to vaccinate, i.e., they had either made or were planning to make an appointment to vaccinate or were waiting for alternate vaccine approval. However, 19% (630/3,390) of patients remained unvaccinated on follow up.

One in four older adults (*n* = 2,138) expressed a desire to seek vaccine information. These adults were ambivalent and required clinical advice. This group of older adults acknowledged the need to protect against the COVID-19 but were hesitant due to lack of clear information on vaccine choices and effectiveness vis-a-vis with other preventive measures. About two-thirds (67%, 1,437/2,138) of this subgroup of adults had taken up COVID-19 vaccination at the end of the program.

While a quarter of patients (24%, *n* = 2,086) refused vaccination without reason on initial contact, 41% (851/2,086) of them subsequently received at least one dose of the COVID-19 vaccine.

8% (715/8,617) of adults had limited access to vaccination sites, such as those who were homebound, residing in residential homes, or hospitalized. Nevertheless, two-thirds (65%, 455/715) of them in this group had received at least one dose of the COVID-19 vaccine by 27 October 2021.

A minority of adults (3%, 288/8,617) perceived risk of adverse effect from vaccinations. These adults were concerned COVID-19 vaccination may worsen their medical condition or trigger an allergic reaction. At the end of the program, 50% (144/288) of them had initiated or completed their vaccination against COVID-19.

65.5% (5,646/8,617) and 64.6% (7,436/11,511) patients in phase I and phase 2, respectively, a total of 60.4% (13,082/21,663), had received at least one dose of COVID-19 vaccine 3 months after the initiation of the COVE program.

**Figure 2:**
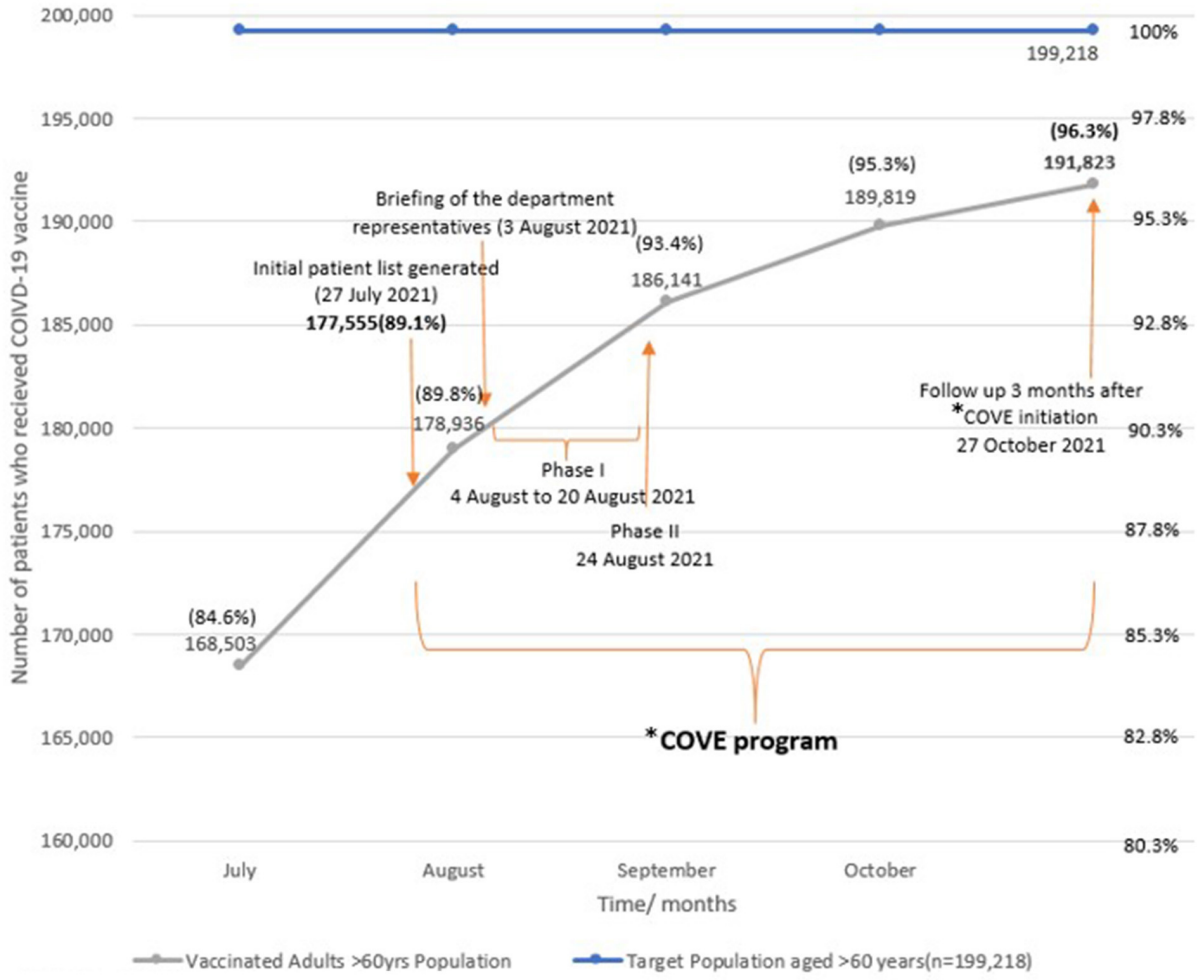
Timeline of COVE program. *COVE, COVID-19 Vaccination in Elderly.

The percentage of patients receiving at least one dose of the COVID-19 vaccine improved from 89.8 to 96.3% over 3 months (27 July 2021 to 27 October 2021), from COVE program initiation to their follow up 3 months later (Figure 2).

## Discussion

This COVE program was designed to acknowledge the potential adverse effects of the vaccine, rectify misinformation and beliefs relating to conspiracy theories, while emphasizing the benefits and social gains of increasing personal immunity against the virus. This person-centric proactive approach has achieved a certain degree of success by increasing the COVID-19 vaccine uptake among the unvaccinated older persons by 60.4% (*n* = 13,082).

During the phone calls, while most of the older adults expressed their intention to vaccinate (39%, *n* = 3,390) or to seek further information (25%, *n* = 2,138), a minority of patients had perceived barriers to access (8%, *n* = 715) or perceived risk of an adverse effect of vaccination (3%, *n* = 288). It was concerning that a quarter (24%, *n* = 2,086) of the adults rejected vaccination without a valid reason.

Toward the end of July 2021, at the time of the COVE program initiation, 12% of the adults in Singapore aged 60–69 years and 20% of the adults older than 70 remained unvaccinated (21). The data is consistent with adults in the age groups that remained unvaccinated in the SHP registry during the same period. According to the diffusion of innovation theory, in any general population, subgroups of innovators, early adopters, early majority and late majority often embrace new changes early. In SHP, 89% of the target recipients were vaccinated in the first few months of the national COVID-19 vaccination program launch. The remaining minority of people (11%), known as “laggards,” are skeptical of change. They are the most challenging group to adopt new changes (29). While the media publicity tends to target the general population, an active personal and targeted approach, like the COVE program, was needed to get the buy-in from this subgroup (22).

Communication to provide relevant information in a way which is easily understood by the older adults and rectifying misinformation improved uptake in COVID-19 vaccination programs (27). A quarter of older adults contacted had gaps in their understanding of the vaccine when contacted by the healthcare team members. The Health Belief Model (HBM) posits that interventions will facilitate behavior change if they successfully address perceived barriers, threats, benefits, self-efficacy and provide cues to action (30, 31).

The COVE program attempted to utilize the HBM constructs to promote COVID-19 vaccine acceptance in these older adults. In the program, healthcare staff spoke to the individual patient in a familiar language or dialect. The intent was to allay fears and raise their awareness of the severity and susceptibility to the infection and benefits of the COVID-19 vaccination. The caller can also direct the patient to trusted information portals to provide the individual with cues to action and to proceed with the COVID-19 vaccination. This simple, step-by-step approach seems to be effective in guiding the older adults to access the healthcare system for their COVID-19 vaccination.

Globally primary care providers have played an essential role in driving COVID-19 vaccine acceptance (32–34). In a national health surveillance survey in Singapore, an estimated 38.4% of residents indicated that they go to a regular family doctor or general physician (35). The long-standing relationship enables patients to trust the recommendation for vaccination by their family physicians. The program driven by the ancillary healthcare workers had supported the physicians to increase the COVID-19 vaccination among the older adults.

One in five adults (19%) who self-reported making plans to vaccinate remained unvaccinated after 3 months of follow up. They could be unwell or had contracted COVID-19 infection, delaying their COVID-19 vaccination. A surge of cases was seen locally during the COVE program (36). Individual medical records relating to hospitalization were not traced due to local privacy regulations. In addition, the older adults could have been influenced by a wide array of anti-vaccination social reports, misinformation or hearsay from their peers or family members (37, 38). A randomized control trial conducted in UK and USA had also reported that misinformation induced a significant decline in the intent to vaccinate among those who stated they would accept a vaccine (39). The Singapore government actively screen media and local websites to mitigate the spread of falsehoods regarding COVID-19 infection and vaccination (40).

A minority (8%, *n* = 715) of the patients had perceived barriers to access. Patients with physical disabilities, homebound or bedbound, may have been unable to go to the vaccination centers. Physical access to vaccination clinics has been identified as a potential barrier to COVID-19 vaccination (41). Arranging transportation and other infrastructural enhancement at the vaccination sites are recommended to improve accessibility to people with disabilities (41). The Singapore government organized outreach programmes like mobile vaccination teams and home vaccination schemes to address limitations to access (42, 43).

The older adults in the program delayed their COVID-19 vaccination because they perceived it could worsen specific medical conditions and concluded the vaccine was unsuitable for them. They were worried about allergic reactions triggered by the COVID-19 vaccine due to prior adverse experience with drug allergy or personal history of atopy. Fear of allergic reaction and worsening of atopy has driven vaccine hesitancy for vaccines (44, 45), even though studies have failed to find a link between vaccination and the risk of atopy (46, 47). While serious allergic reactions to COVID-19 vaccines are rare, the healthcare teams have been trained to manage them promptly at the vaccination sites and to evacuate affected patients to hospitals if necessary (48). Health care professionals must take precautions not to unintentionally generate misinformation that results in COVID-19 vaccine hesitancy (49). Other measures to allay the fears include referral to allergists for those with prior anaphylaxis or severe allergic reactions (50). The local government has also arranged special insurance coverage for those who are hospitalized for severe allergic reaction, so that these patients are not burdened by additional healthcare expenditures (48).

A recent systematic review reported up to 27% older adults were unwilling to be vaccinated against COVID-19 (23). As of 30 December 2021, no population in any country had attained 100% COVID-19 vaccination (51). Hence, continuous efforts are needed to identify patients who miss COVID-19 vaccinations so that personalized measures can be taken to address their individual barriers over multiple healthcare visits. Information technology and other digital health measures can be deployed to assist healthcare workers in this endeavor. A meta-analysis of randomized control trials showed that computer reminders in an ambulatory setting improved pneumococcal and influenza vaccination delivery to adults (50).

The new Omicron VOC has become the predominant variant globally by the end of year 2021 and dominant even among those vaccinated. Omicron poses high risk to older adults, and booster shots are highly advisable (52, 53). Thus, it is crucial to proactively engage this population for repeated vaccination to maintain their immunity against any new emerging VOC. Concurrently, vaccination campaigns will be organized regularly to boost preventive health and herd immunity in the community healthcare system.

### Strengths and limitations

A multidisciplinary team who volunteered and supported the COVE program was a strength of this program. Involving non-clinical staff in the program supported the medical staff in their clinical work.

The COVE program was limited by the absence of a control arm to compare its effectiveness against usual public health measures. Thus, measuring the effect of the COVE program on directly changing the older adults' decision regarding COVID-19 vaccine uptake is challenging. However, ethical considerations may deter such a study design. Its implementation was also hindered by failure to update the patient registry in the institution due to change of addresses, contact numbers, or overseas travel among patients. Furthermore, an estimated 21.6% of Singaporean residents aged 65 years and older live alone (36), and 40% of Singapore residents aged >75 years do not use a handphone (37). Partnering social service providers and community healthcare partners can aid to identify and vaccinate disadvantaged and socially isolated patients in the general population.

## Conclusion

The COVE program improved vaccination uptake of adults >60 years in SHP, enabling 96.3% of the older adults in the SHP registry and 60.4% of those targeted in the intervention to receive at least one dose of vaccine against COVID-19 by the end of the program. Perceived adverse reaction to the vaccine, limited access to the vaccination sites, inadequate or misinformation on the COVID-19 vaccine are common barriers among the older adults. Direct contact and clarity in communication using mutually understood languages can boost their COVID-19 vaccination. Consultation with allergists and direct access to allergy clinics are ways to manage patients' fear of allergic reactions to COVID-19 vaccines. Future programs should engage the community partners to identify socially isolated and unreachable older adults to ensure an all-inclusive vaccination program.

### Acknowledgment of any conceptual or methodological constraints

This community case study is an *ad-hoc* intervention to elevate the COVID-19 vaccine uptake among a high-risk but heterogeneous subset of the population. The study is limited by a lack of a control group to compare the effectiveness of the COVE program due to urgency to scale the COVID-19 vaccination among the older adults amidst emerging variants of concern. The results were reported as a difference in their vaccine uptake before and after the COVE implementation. The change in mindset by these older adults that is directly attributed by the brief phone intervention by the healthcare workers remains unclear. While a script was provided to induct the healthcare workers, the content of their phone conversation was expected to vary due to the range of concerns raised by these older adults who were of different demographic profiles, posing challenges to their fidelity of the intervention.

## Data availability statement

The original contributions presented in the study are included in the article/supplementary material, further inquiries can be directed to the corresponding author/s.

## Ethics statement

Ethical review and approval was not required for the study on human participants in accordance with the local legislation and institutional requirements. Written informed consent for participation was not required for this study in accordance with the national legislation and the institutional requirements.

## Author contributions

MJ and WA provided the data. QT and YK analyzed the data. AM and YW wrote the first draft of the manuscript. NT actively contributed to revise the manuscript. WT, GE, CN, and CL provided constructive comments to improve and finalize the manuscript. All authors have read and agreed to the published version of the manuscript.

## Funding

The publication of this study is funded by SingHealth Polyclinic Research Department.

## Conflict of interest

The authors declare that the research was conducted in the absence of any commercial or financial relationships that could be construed as a potential conflict of interest.

## Publisher's note

All claims expressed in this article are solely those of the authors and do not necessarily represent those of their affiliated organizations, or those of the publisher, the editors and the reviewers. Any product that may be evaluated in this article, or claim that may be made by its manufacturer, is not guaranteed or endorsed by the publisher.
